# Patient-specific three-dimensional planning for surgical approach selection in parapharyngeal space lesions: development of the Vall d’Hebron 3D-PPS Algorithm

**DOI:** 10.3389/fonc.2026.1954820

**Published:** 2026-09-21

**Authors:** Pablo Vergés Fort, Juan Fernando Fuentes Cabrera, Sílvia Naches Solsona, Laura Grau deDiego, Laura Escot Garcia, Juan Carlos Tortajada Bustelo, Jose Miguel Rodríguez Sánchez, David Virós Porcuna

**Affiliations:** 1 Otolaryngology Head and Neck Department, Vall d’Hebron Barcelona Hospital Campus, Barcelona, Spain; 2 Universitat Autònoma de Barcelona, UD Vall Hebron, Barcelona, Spain; 3 Vall d’Hebron Research Institute (VHIR), Barcelona, Spain

**Keywords:** 3D planning, head and neck surgery, parapharyngeal space (PPS), parapharyngeal tumor, salivary gland tumours, transoral robotic surgery (TORS), transoral robot assisted surgery, virtual surgical planning

## Abstract

**Introduction:**

Surgical management of parapharyngeal space (PPS) lesions remains challenging because of their deep anatomical location and close relationship to major neurovascular structures. Although patient-specific three-dimensional (3D) reconstruction improves anatomical visualisation, standardised methods for incorporating this information into surgical approach selection remain poorly defined. We developed the Vall d’Hebron 3D-PPS Workflow and Algorithm to integrate patient-specific anatomy into preoperative decision-making.

**Methods:**

Prospective observational study including 17 consecutive patients with PPS lesions treated at a tertiary referral centre. All patients underwent patient-specific 3D virtual planning integrating CT and MRI datasets. The primary outcome was the impact of the Vall d’Hebron 3D-PPS Workflow on surgical approach selection. Secondary outcomes included postoperative complications, surgical margins or completeness of lesion resection, length of hospital stay and surgeon-reported usefulness of the 3D models.

**Results:**

Surgical treatment consisted of transoral robotic surgery (TORS) in seven patients (41.2%), transcervical surgery in four (23.5%), combined external plus TORS in four (23.5%) and transcervical–transparotid surgery in two (11.8%). Patient-specific 3D planning modified the initially planned surgical approach in four patients (23.5%). Surgeons rated the models as very useful (Likert 5) in 76.5% of cases and useful (Likert 4) in the remaining 23.5%. Complete lesion resection or negative surgical margins were achieved in 82.4% of patients. Postoperative complications occurred in two patients (11.7%), and no mortality was observed.

**Conclusion:**

The Vall d’Hebron 3D-PPS Workflow complements conventional imaging by integrating patient-specific anatomy into a structured preoperative decision-making process for surgical approach selection in PPS lesions.

## 1 Introduction

Surgical management of parapharyngeal space (PPS) lesions remains one of the most technically demanding challenges in head and neck surgery. Although these lesions account for less than 1% of all head and neck neoplasms, they comprise a heterogeneous group of tumours and selected vascular abnormalities arising within a deep anatomical compartment closely related to the internal carotid artery (ICA), internal jugular vein, and lower cranial nerves. This complex anatomy makes surgical approach selection particularly demanding, requiring an optimal balance between adequate surgical exposure, preservation of neurovascular structures, and functional outcomes (1, 2).

The PPS is classically described as an inverted pyramid extending from the skull base to the hyoid bone and divided into prestyloid and retrostyloid compartments, each containing distinct anatomical structures that give rise to characteristic pathological entities (1). Most lesions are benign, with pleomorphic adenomas and schwannomas representing the most frequent histological diagnoses (3). Because this anatomical space allows considerable lesion growth before symptoms develop, tumours are frequently diagnosed only after reaching a substantial size, further increasing the complexity of surgical planning and resection.

Traditionally, PPS lesions have been managed through open surgical approaches, including transcervical, transparotid and transmandibular techniques, which provide adequate exposure and vascular control but are associated with significant morbidity, including extensive soft tissue dissection, visible cervical scars, cranial nerve injury and prolonged postoperative recovery (2, 4). The introduction of transoral robotic surgery (TORS) has expanded the surgical armamentarium by providing a minimally invasive alternative for selected lesions, particularly those located within the prestyloid compartment (5–7). The superior visualisation and instrument dexterity offered by robotic surgery have increased the feasibility of transoral access; however, they have also highlighted the importance of appropriate patient selection. As TORS indications have expanded, determining which lesions can be safely approached through a transoral corridor has become increasingly relevant (8).

Advances in cross-sectional imaging have substantially improved preoperative evaluation of PPS lesions. More recently, patient-specific three-dimensional (3D) reconstruction has enabled intuitive visualisation of the spatial relationships between tumours and surrounding anatomical structures, facilitating surgical planning in anatomically complex regions (9–11). Nevertheless, despite these technological advances, the integration of 3D planning into surgical decision-making remains poorly defined. Current evidence primarily focuses on improving anatomical visualisation, whereas standardised methods for translating patient-specific 3D information into reproducible surgical approach selection are still lacking.

To address this unmet need, we developed a structured patient-specific preoperative planning workflow integrating conventional imaging, three-dimensional reconstruction and multidisciplinary assessment. Within this workflow, we designed the Vall d’Hebron 3D-PPS Algorithm, a practical decision-support strategy intended to assist surgical approach selection according to individual tumour anatomy and its relationship with surrounding vascular structures. The novelty of the present study does not lie in three-dimensional reconstruction itself, but in translating patient-specific anatomical information into a reproducible anatomy-driven clinical decision-support algorithm for surgical approach selection.

The present prospective study evaluates the feasibility and clinical impact of this strategy in a consecutive series of patients with PPS lesions, with particular emphasis on its influence on surgical decision-making and approach selection.

## 2 Methods

### 2.1 Study design and setting

This prospective observational study included 17 consecutive patients with PPS lesions treated between December 2024 and December 2025 at Hospital Universitari Vall d’Hebron (Barcelona, Spain), a tertiary academic referral centre with a dedicated in-house 3D laboratory formally approved by the relevant regional authorities [PR(AG)157/2025]. The study was conducted in accordance with the Declaration of Helsinki. All patients provided written informed consent for both surgical treatment and inclusion in the 3D planning protocol. Consecutive patient inclusion was adopted to minimise selection bias and to prospectively evaluate the clinical applicability of the proposed workflow under routine clinical practice.

### 2.2 Patient selection

Adult patients (≥18 years) with radiologically confirmed PPS lesions scheduled for surgical treatment were consecutively included. Exclusion criteria were: (i) incomplete preoperative imaging datasets; (ii) absence of 3D planning; or (iii) non-surgical management.

Fine-needle aspiration cytology (FNAC) was performed transorally or transcervically whenever considered clinically appropriate. However, preoperative histological diagnosis was not incorporated as a primary determinant of surgical approach selection. Histology was used to guide oncological planning, anticipated resection margins and vascular preparedness in suspected malignant lesions, whereas the selection of the surgical corridor was primarily based on patient-specific anatomical characteristics.

### 2.3 Preoperative assessment

#### 2.3.1 Conventional imaging

All patients underwent contrast-enhanced CT with ≤1 mm slice thickness and MRI (1.5T or 3T). Contrast-enhanced CT provided detailed assessment of osseous anatomy and vascular structures, whereas MRI was primarily used for lesion characterisation and delineation of soft tissue interfaces.

#### 2.3.2 Three-dimensional virtual surgical planning

Three-dimensional planning followed the institutional Vall d’Hebron 3D-PPS Workflow, consisting of three sequential phases: image reconstruction, anatomical segmentation, and integrated multidisciplinary analysis.

DICOM datasets from CT and/or MRI were imported into a dedicated 3D planning workstation. Serial two-dimensional slices were converted into volumetric datasets (**Figure 1**).

**Figure 1:**
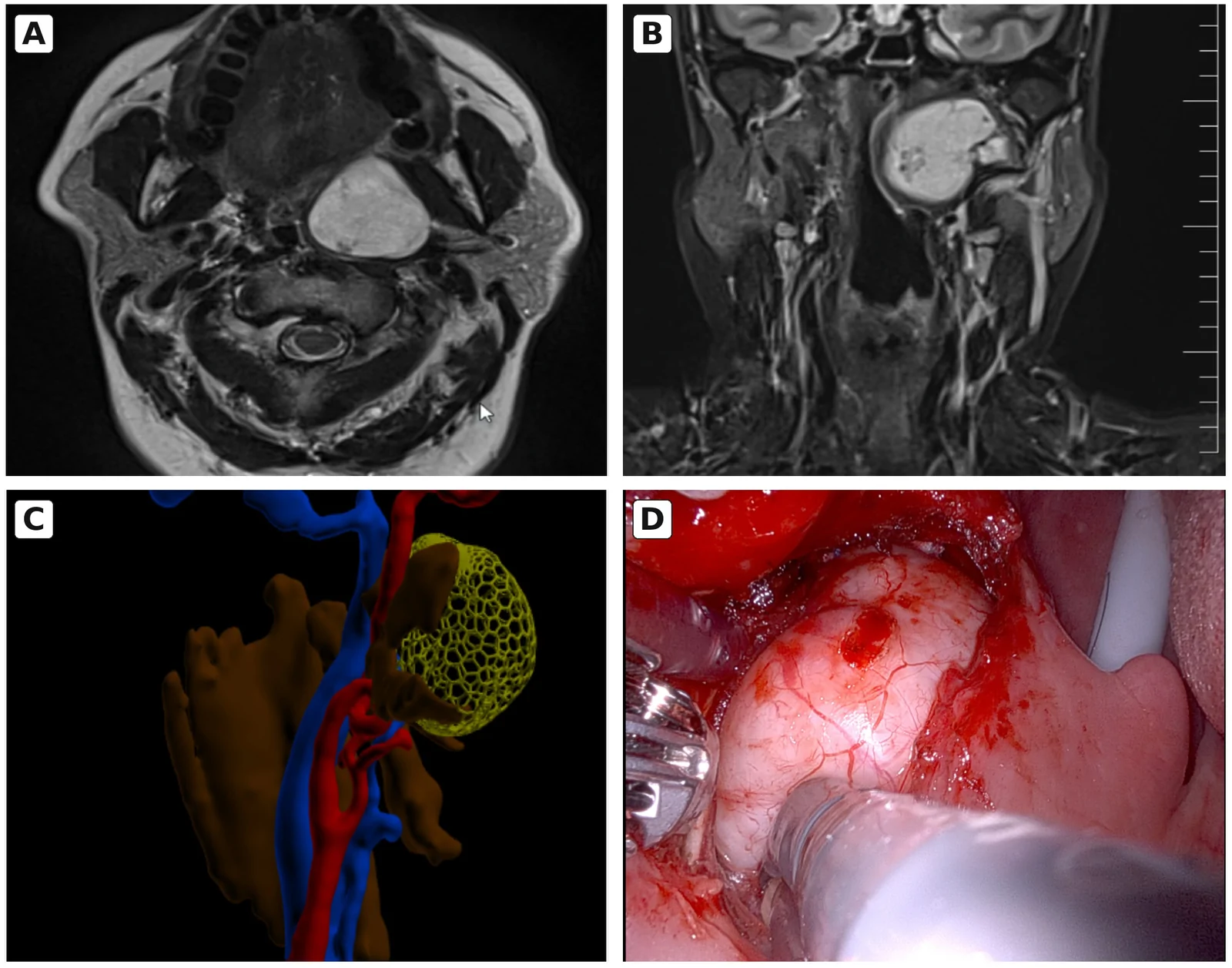
Preoperative study magnetic resonance imaging illustrating tumour localisation within the parapharyngeal space. Patient-specific three-dimensional reconstruction used for preoperative planning, demonstrating tumour relationships with the internal carotid artery, pterygoid muscles, and adjacent anatomical structures. Intraoperative view of transoral robotic surgical resection of a parapharyngeal space tumour.

Semi-automated segmentation was performed by trained biomedical engineers, with manual refinement as required. This segmentation protocol was standardised for all patients (**Table 1**).

**Table 1:** Anatomical structures included in the segmentation protocol.

Category	Structures segmented
Lesion	Lesion volume and relevant anatomical boundaries
Vascular	Internal carotid artery (ICA), external carotid artery and branches, internal jugular vein
Musculoskeletal	Pterygoid muscles, styloid process and associated musculature (styloglossus, stylopharyngeus, stylohyoid), mandible, prevertebral musculature
Glandular	Deep lobe of the parotid gland
Airway/other	Airway lumen, additional structures at surgeon’s request

ICA, internal carotid artery.

Finally, a joint review session was held with a dedicated head and neck radiologist to ensure accurate identification of critical structures.

**Table 2** summarises all preoperative assessment variables collected across imaging and 3D planning domains.

**Table 2:** Variables included in the Vall d’Hebron 3d-PPS workflow.

Domain	Variable	Assessment method
Conventional imaging (CT/MRI)	Lesion size and volume	CT ≤1 mm/MRI 1.5–3T
	PPS compartment (prestyloid/retrostyloid)	CT + MRI
	Craniocaudal extension and skull base proximity	CT + MRI
	Relationship to deep parotid lobe	MRI
	Displacement or contact with major vessels	CT angiography/MRI
3D virtual planning	Lesion-ICA spatial relationship	3D model
	Medial vs. lateral accessibility	3D model
	Feasibility of transoral corridor	3D model
	Identification of high-risk vascular zones	3D model
	Styloid process and muscular relationships	3D model
	Anticipated dissection planes	3D model
Surgeon assessment	Perceived utility of 3D model (Likert 1–5)	Surgeon-reported questionnaire
	Impact on approach selection vs. conventional imaging	Pre/post-planning comparison

ICA, internal carotid artery; CT, computed tomography; MRI, magnetic resonance imaging.

Segmented structures were rendered into patient-specific virtual 3D models with interactive visualisation capabilities (rotation, magnification, transparency adjustment). Digital models were systematically reviewed by the surgical team. Physical 3D printing was performed only in selected anatomically complex cases, at the discretion of the operating surgeon.

### 2.4 Integration the Vall Hebron 3D-PPS algorithm in surgical decision-making

Following completion of patient-specific reconstruction, all cases underwent a second multidisciplinary review integrating clinical information, conventional imaging and three-dimensional anatomical assessment.

The Algorithm was designed as a hierarchical anatomy-driven clinical decision-support algorithm rather than a single-parameter decision tree.

The relationship between the lesion and the internal carotid artery constituted the initial decision point because it primarily determined the feasibility and safety of a purely transoral robotic corridor. Subsequently, additional patient-specific anatomical variables were sequentially incorporated into surgical planning, including:

Lateral extension beyond the medial pterygoid muscle; Involvement or origin from the deep lobe of the parotid gland; Cranial extension towards the skull base; Tumour size; Anticipated need for vascular control.

These variables were interpreted hierarchically rather than independently.

Large tumours (>6 cm) were not considered a contraindication to TORS per se but frequently required combined approaches because of their greater lateral extension and increased need for vascular exposure.

Likewise, lesions demonstrating suspected carotid involvement were not automatically excluded from minimally invasive surgery; instead, the algorithm facilitated preoperative planning of vascular control strategies, multidisciplinary coordination and selection of the safest surgical approach.

Surgical approach was categorised as: (i) TORS; (ii) open lateral approach (transcervical, transcervico-transparotid); or (iii) combined external + TORS. The final surgical approach was determined using the Vall d’Hebron 3D-PPS Algorithm (**Figure 2**) after integration of clinical findings, conventional imaging and patient-specific 3D reconstruction during multidisciplinary review.

**Figure 2:**
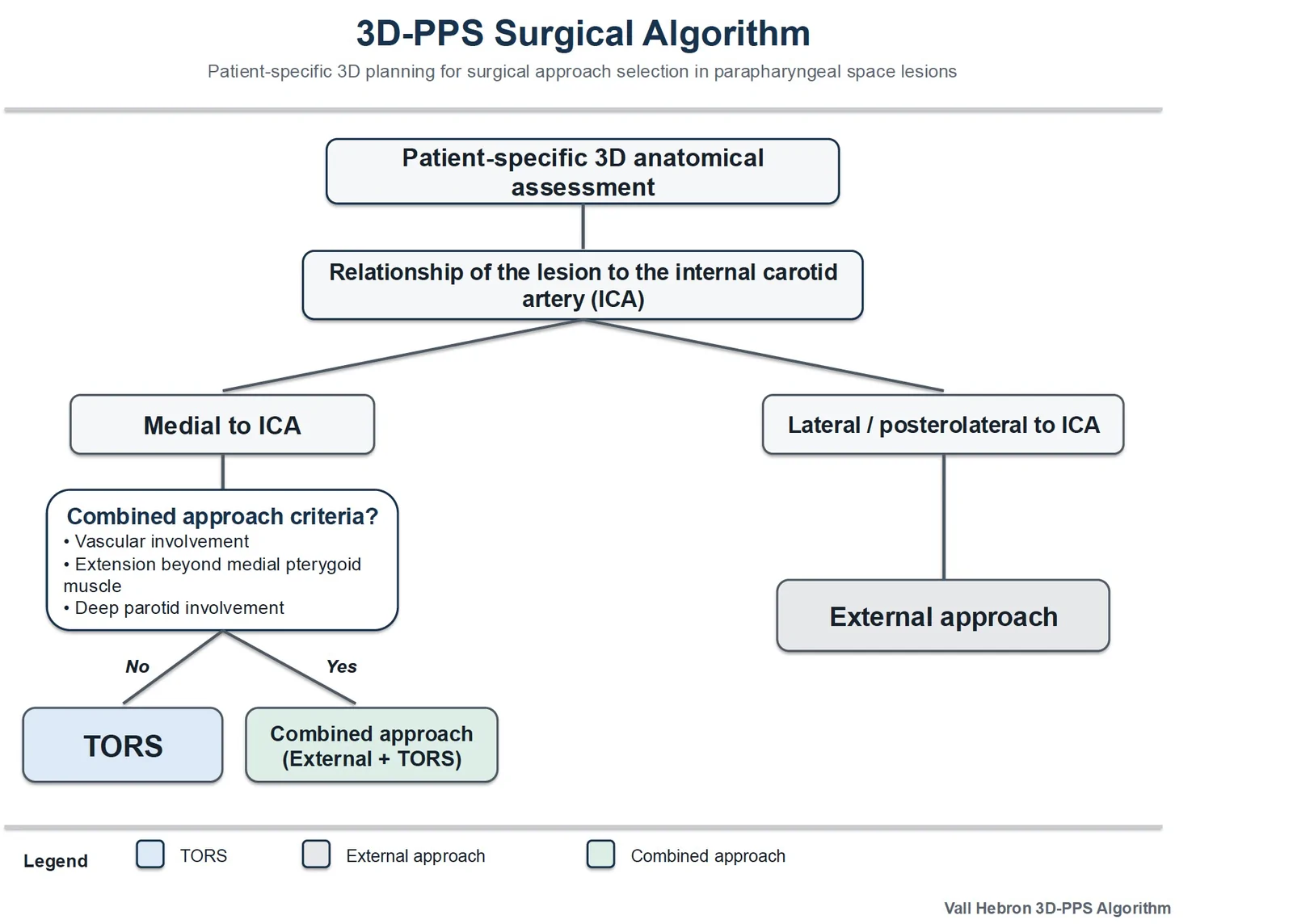
The Vall d’Hebron 3D-PPS Algorithm for surgical approach selection.

Importantly, the algorithm was designed to optimise anatomical accessibility rather than tumour biology. Histological diagnosis therefore influenced oncological planning but did not constitute the principal determinant of surgical corridor selection.

The proposed workflow was designed to standardise preoperative anatomical assessment rather than replace clinical judgement.

### 2.5 Surgical technique

TORS was performed using the da Vinci Xi^®^ robotic system (Intuitive Surgical, Inc., Sunnyvale, CA) with the Feyh-Kastenbauer retractor modified by Weinstein-O’Malley (Olympus Corp), docked at the patient’s left side. Surgery was performed in a slightly tilted head-up position. A single dose of dexamethasone 4–8 mg was administered intravenously to prevent postoperative airway oedema. Prophylactic tracheostomy was not routinely performed; patients with postoperative oedema or uncertain airway safety remained intubated for 24 hours, with temporary tracheostomy reserved for failure to improve. The initial incision was made in the pterygomandibular raphe; after dissection of the palatoglossus and constrictor muscles, the prestyloid PPS was exposed by blunt dissection. When lateral extension was present, the medial pterygoid muscle was partially incised to expand the axial corridor. A 0-degree scope was used for the middle and inferior PPS; a 30-degree scope for the superior compartment (8).

Open approaches included transcervical and cervico-transparotid techniques according to lesion characteristics. The transcervical approach uses a 4-cm transverse skin incision; after identification of the posterior digastric belly and stylohyoid muscle, the stylomandibular ligament is divided to improve exposure. The cervico-transparotid approach additionally identifies the facial nerve and provides access to the deep parotid lobe and lateral prestyloid space. Intraoperative facial nerve monitoring (NIM system, Medtronic Corp., four channels) was used in all open and combined cases. All procedures aimed at complete lesion resection with preservation of neurovascular structures whenever oncologically or anatomically feasible.

In combined procedures, the surgical dissection was performed in a sequential and complementary fashion, progressing either from the transoral robotic approach towards the cervical field or conversely from the transcervical approach towards the transoral corridor, depending on the anatomical characteristics of the lesion and intraoperative requirements.

For patients undergoing transoral robotic surgery, postoperative nasogastric tube placement formed part of the institutional postoperative protocol rather than reflecting clinically significant dysphagia. The tube was routinely maintained for approximately 48 hours to protect the pharyngeal closure during the early healing phase before resumption of oral feeding.

### 2.6 Outcome measures and statistical analysis

The primary outcome was the clinical impact of patient-specific three-dimensional planning on surgical approach selection, defined as any modification of the initially proposed surgical strategy following application of the Vall d’Hebron 3D-PPS Algorithm.

- Secondary outcomes included:
- postoperative complications;
- completeness of lesion resection or surgical margins;
- hospital stay;
- duration of nasogastric tube;
- surgeon-perceived usefulness of the 3D model.

Surgical margins were considered free if complete extracapsular dissection was achieved in benign tumours, or a ≥1 mm tumour-free margin in malignant tumours. In the vascular lesion, complete resection of the aneurysm was considered equivalent to successful complete lesion removal. The usefulness of patient-specific three-dimensional planning was independently assessed immediately after surgery by the primary operating surgeon using a five-point Likert scale (13). Descriptive statistics were used; categorical variables compared using Fisher’s exact test and continuous variables using the Mann-Whitney U test (Stata v14.2, StataCorp LLC). Statistical significance was set at P ≤ 0.05.

## 3 Results

### 3.1 Patient characteristics

Seventeen consecutive patients underwent surgical treatment for parapharyngeal space lesions during the study period and were prospectively included in the analysis. Median age was 49.5 years (mean 50.8; range 26–74), with a female predominance. Lesion diagnoses and surgical outcomes by approach are detailed in **Table 3**. Pleomorphic adenoma was the most common diagnosis (n=8, 47.1%)), followed by schwannoma (n=2, 11.8%). Less common lesions included oncocytoma, secretory carcinoma, lymphangioma, giant cell tumour, carotid paraganglioma, vagal paraganglioma, and one parapharyngeal internal carotid artery aneurysm.

**Table 3:** Histopathological diagnoses, and surgical approach.

Histological diagnosis/lesion type	n	Surgical approach
Pleomorphic adenoma	8	TORS (4)/Combined (3)/TC (1)
Schwannoma (CN IX)	2	TORS (1)/TC(1)
Oncocytoma	1	TC-TP
Secretory carcinoma	1	Combined
Lymphangioma	1	TORS
Giant cell tumour	1	TORS
Carotid paraganglioma	1	TC
Vagal paraganglioma	1	TC
Parapharyngeal internal carotid artery aneurysm	1	TC-TP
**Total**	**17**	**TORS n=7, Combined n=4 TC n=4, TC-TP n=2**

TORS, transoral robotic surgery; TC, transcervical; TC-TP, transcervical-transparotid;.

Bold values indicate the total number of cases for each surgical approach.

Patient-specific three-dimensional reconstruction was successfully performed in all patients without technical failures, allowing complete anatomical evaluation before multidisciplinary surgical planning.

Surgical treatment consisted of transoral robotic surgery (TORS) in seven patients (41.2%), transcervical resection in four (23.5%), combined external plus TORS in four (23.5%), and transcervical–transparotid surgery in two patients (11.8%) (**Table 3**).

Amongst pleomorphic adenomas, the median tumour size was 49 mm (range, 30–70 mm). Tumours managed by TORS had a median size of 46 mm (range, 30–63 mm), whereas those requiring combined approaches had a median size of 52 mm (range, 35–65 mm). Despite a tendency for larger tumours to require combined approaches, there was substantial overlap in tumour size between groups.

### 3.2 Clinical impact of the Vall d’Hebron 3D-PPS workflow

Application of the Vall d’Hebron 3D-PPS Algorithm resulted in modification of the initially proposed surgical approach in four patients (23.5%) following multidisciplinary review of the patient-specific three-dimensional reconstruction.

The principal anatomical findings responsible for these modifications included identification of significant lateral extension beyond the medial pterygoid muscle, more accurate definition of the lesion’s relationship with the deep lobe of the parotid gland, and improved assessment of the proximity between the tumour and the internal carotid artery. These additional anatomical findings altered the anticipated surgical exposure required to achieve safe tumour resection and therefore modified the final surgical strategy.

Specifically, 3D planning prompted conversion from a transoral to an external approach in cases where vascular proximity limited safe transoral access, enabled preoperative identification and planned exposure of the internal carotid artery during resection (**Supplementary Video 1**), facilitated preoperative organisation of vascular surgical backup in one patient, and anticipated the vascular risk in the patient who subsequently experienced an intraoperative carotid artery rupture, allowing immediate management by the interventional radiology team (**Figure 3**).

**Figure 3:**
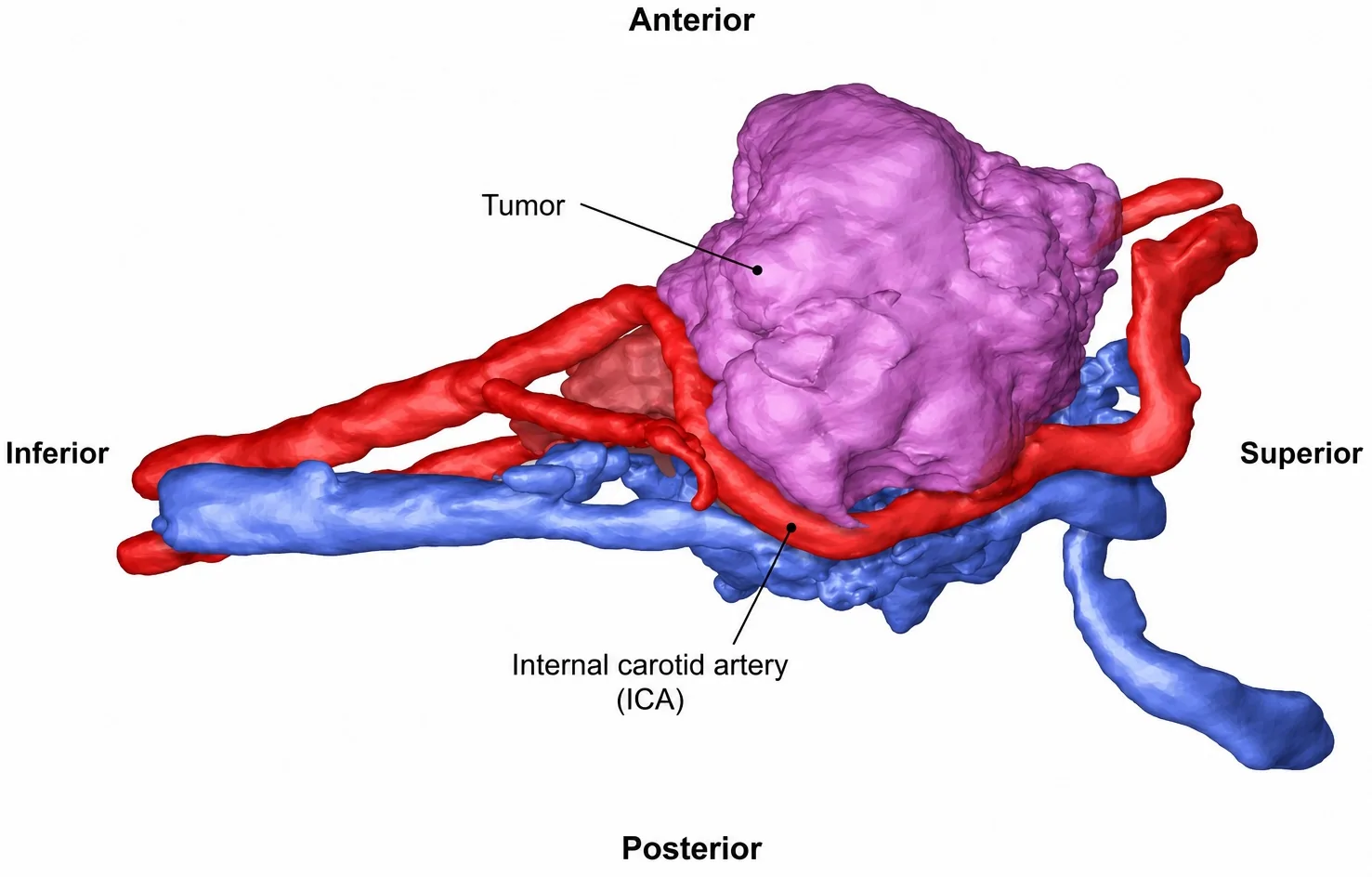
Patient-specific three-dimensional reconstruction illustrating the spatial relationship between the parapharyngeal space tumour and the internal carotid artery (ICA). The reconstruction provides an intuitive visualisation of the tumour–vascular interface, facilitating preoperative assessment of surgical accessibility and selection of the optimal surgical approach. Anatomical orientation (anterior, posterior, superior and inferior) is shown for reference.

The four cases in which the surgical strategy changed after application of the algorithm are summarised in **Table 4**, including the initially planned approach, the anatomical finding responsible for the modification, the final surgical approach selected and the corresponding clinical outcome.

**Table 4:** Modification of surgical strategy due to Vall d’Hebron 3D-PPS Algorithm application.

Case	Histology	Initial planned approach	3D anatomical finding modifying strategy	Final surgical approach	Main reason for change	Outcome
1	Pleomorphic adenoma	TORS	Lateral extension requiring vascular control	Combined TORS + transcervical	Safe vascular control	Complete resection
2	Secretory carcinoma	TORS	Close ICA relationship suggesting possible vascular invasion	Combined TORS + transcervical	Planned vascular preparedness	ICA invasion managed with endovascular support
3	Pleomorphic adenoma	Combined	Tumour located predominantly lateral to the pterygoid corridor and deep to the parapharyngeal fat pad	Transcervical	Inadequate transoral accessibility	Complete resection
4	Pleomorphic adenoma	TORS	Greater lateral extension than expected on conventional imaging	Combined TORS + transcervical	Improved exposure and vascular safety	Complete resection

Following completion of each surgical procedure, the primary operating surgeon independently completed a five-point Likert questionnaire evaluating the perceived usefulness of patient-specific three-dimensional planning. Surgeons rated the patient-specific 3D models as very useful (Likert score 5) in 13 cases (76.5%) and useful (Likert score 4) in the remaining four cases (23.5%).

Overall, three-dimensional reconstruction received high scores regarding anatomical understanding, confidence in surgical planning and intraoperative orientation. The highest ratings were observed in cases presenting complex vascular anatomy or requiring combined surgical approaches.

The most frequently reported benefits were improved understanding of the tumour–vascular relationship and better anticipation of surgical dissection planes.

### 3.3 Surgical outcomes

Complete resection with negative margins (or complete lesion removal in the vascular lesion) was achieved in 14 patients (82.4%). Three patients (17.7%) presented positive surgical margins. One patient had a secretory carcinoma with intraoperative invasion of the internal carotid artery. Although preoperative imaging suggested close contact with the vessel, direct vascular infiltration could only be confirmed intraoperatively. Patient-specific three-dimensional planning had accurately identified this case as presenting increased vascular complexity, allowing preoperative coordination with the endovascular team and immediate availability of vascular rescue strategies during surgery.

The remaining two patients were recurrent pleomorphic adenomas measuring 63 mm and 55 mm, respectively, both managed through combined approaches. In both cases, capsular rupture was identified intraoperatively, requiring widening of the dissection whilst preserving adjacent critical neurovascular structures. Although microscopic surgical margins were positive, neither patient has developed local recurrence after 12 months of follow-up. Overall, incomplete resection was related to tumour biology and local disease characteristics rather than limitations of the selected surgical corridor.

### 3.4 Postoperative outcomes

Median length of hospital stay was 5 days (range 3–13 days).

Nasogastric tube placement was performed in 16 patients (94,1%) according to the institutional postoperative protocol. The tube was routinely maintained for approximately 48 hours during the early postoperative period before resumption of oral intake to help for oropharyngeal proper healing. The tube median duration was 3 days (range 0–22 days, counting the patient without NGT as 0 days).

Postoperative complications rate was 11.7% (n=2) one transient peripheral facial nerve palsy and one internal carotid rupture. Negative surgical margins or complete lesion resection were achieved in 14 patients (82.4%). No mortality was recorded.

## 4 Discussion

Surgical management of parapharyngeal space (PPS) lesions remains technically demanding because surgical approach selection requires balancing adequate surgical exposure with preservation of surrounding neurovascular structures and postoperative function. Although open approaches continue to play an essential role in selected cases, the introduction of transoral robotic surgery (TORS) has expanded the range of minimally invasive options for appropriately selected tumours (5–7). In this context, the principal finding of the present study is that patient-specific three-dimensional (3D) planning can be systematically incorporated into preoperative assessment through a structured workflow, providing clinically relevant information that modified the initially planned surgical approach in almost one-quarter of patients. Rather than proposing a new imaging technology, this study proposes a structured method for incorporating patient-specific anatomy into surgical decision-making.

Conventional CT and MRI remain the cornerstone of preoperative evaluation of PPS lesions (1, 2). They accurately characterise lesion size, compartment and relationship to adjacent structures; however, interpretation of the spatial relationship between the lesion, the internal carotid artery (ICA), surrounding musculature and potential surgical corridors frequently relies on individual surgical experience. In our series, patient-specific 3D reconstruction complemented conventional imaging by facilitating a more intuitive understanding of these complex anatomical relationships, particularly in lesions with borderline indications for a transoral approach. Rather than replacing conventional imaging, the proposed workflow integrates anatomical information into a structured decision-making process that supports surgical approach selection.

Previous reports have demonstrated the value of virtual surgical planning and three-dimensional reconstruction for improving anatomical understanding in complex head and neck procedures (9–11). More recently, these technologies have also been applied to PPS surgery, mainly to facilitate preoperative visualisation and improve surgeon confidence during transoral procedures.9, 10] However, most published studies have focused on the technical aspects of image reconstruction, whereas less attention has been paid to how this additional anatomical information can be translated into a reproducible strategy for surgical approach selection. In the present study, the Vall d’Hebron 3D-PPS Algorithm was developed as a practical framework to integrate patient-specific anatomy into routine multidisciplinary decision-making rather than as an isolated planning tool.

The greatest clinical benefit of this strategy appears to arise in cases where conventional imaging alone does not clearly define the safest surgical corridor. Three-dimensional planning modified the initial surgical strategy in 23.5% of patients. Rather than representing changes in surgical preference, these modifications reflected improved understanding of tumour anatomy and its relationship with adjacent structures. Identification of previously underestimated lateral extension, more accurate definition of deep lobe parotid origin or recognition of increased vascular complexity resulted in selection of safer or more appropriate surgical approaches. These findings suggest that the principal value of patient-specific three-dimensional planning may reside not simply in enhancing anatomical visualisation but in improving the quality and reproducibility of surgical decision-making. The patient with intraoperative internal carotid artery invasion further illustrates this concept. Three-dimensional reconstruction did not predict vascular infiltration with certainty, nor was this the purpose of the algorithm. Instead, identification of close anatomical proximity between the tumour and the carotid artery prompted preoperative anticipation of a high-risk vascular scenario, multidisciplinary planning and immediate availability of endovascular support. Consequently, the algorithm should not be interpreted as a tool capable of predicting oncological behaviour, but rather as a strategy for anticipating surgical complexity and facilitating appropriate preparation for potentially hazardous intraoperative situations.

Appropriate patient selection remains fundamental for successful TORS. Systematic reviews have consistently demonstrated favourable functional outcomes and reduced morbidity following transoral resection of selected PPS tumours whilst emphasising the importance of careful patient selection (5–7, 12–14). Particular attention should be paid to pleomorphic adenomas, in which capsular violation may compromise long-term local control (15, 16). Similarly, previous publications (17, 18), identified several favourable anatomical characteristics for minimally invasive surgery.

Interestingly, although larger pleomorphic adenomas tended to require combined approaches, tumour size alone did not reliably discriminate between patients managed with TORS and those requiring combined surgery because of the substantial overlap observed between groups. These findings further support the tumour size contributes to decision-making but remains secondary to the other analyzed characteristics.

One of the principal conceptual strengths of the proposed algorithm is that it does not rely on a single anatomical parameter. Although the relationship between the lesion and the internal carotid artery represents the initial decision point because of its major implications for surgical safety, operative planning ultimately results from the sequential integration of multiple patient-specific anatomical variables. Lateral extension beyond the medial pterygoid muscle, deep lobe parotid involvement, cranial extension towards the skull base and anticipated vascular requirements all contribute to the final decision regarding the most appropriate surgical corridor. This hierarchical interpretation better reflects the complexity of real clinical decision-making than simplified classifications based on individual radiological findings.

Importantly, the proposed algorithm was intentionally designed to optimise anatomical accessibility rather than tumour biology. Histopathological diagnosis remains essential for defining oncological strategy, expected resection margins and adjuvant treatment; however, surgical corridor selection primarily depends on anatomical relationships rather than histological subtype. This distinction is particularly relevant because malignant lesions may remain suitable for minimally invasive surgery provided that anatomical conditions are favourable, whereas some benign tumours may require combined or conventional approaches because of their anatomical extension.

Beyond individual surgical planning, the algorithm also proved valuable as a multidisciplinary communication tool. The patient-specific three-dimensional models facilitated discussion between surgeons, radiologists and biomedical engineers, providing a shared understanding of complex anatomy before surgery. This educational component may become increasingly relevant as robotic surgery continues to expand and more centres incorporate advanced virtual planning into routine clinical practice.

An additional contribution of this work is the proposal of a standardised preoperative workflow. Existing algorithms for PPS surgery are largely based on tumour compartment, histology and vascular relationships (4, 18, 19). Our workflow incorporates these established principles whilst adding patient-specific three-dimensional anatomical assessment as an additional layer of information during multidisciplinary planning. Rather than proposing a new imaging technology, this strategy provides a structured method for integrating patient-specific anatomy into surgical decision-making. Although external validation is required, such an approach may improve consistency in surgical planning and facilitate implementation across centres with different levels of experience.

Patient-specific 3D reconstruction may also provide the basis for future developments in image-guided surgery. As deformable models, augmented reality and intraoperative navigation continue to evolve, individualised anatomical models could facilitate integration between preoperative planning and intraoperative guidance, particularly in soft-tissue environments where conventional navigation remains limited (11, 20).

Several limitations should be acknowledged. First, this is a prospective single-centre study including a relatively limited number of patients, reflecting the rarity of PPS tumours. Consequently, external validation in larger multicentre cohorts will be necessary before widespread implementation can be recommended. Second, although patient-specific three-dimensional reconstruction was successfully achieved in all cases, its application currently requires dedicated segmentation software, specialised technical expertise and close collaboration between surgeons, radiologists and biomedical engineers, which may limit immediate adoption in lower-volume institutions. Finally, the algorithm was developed within a high-volume tertiary referral centre with extensive experience in robotic surgery and advanced head and neck procedures; therefore, its generalisability should be interpreted within this clinical context.

Nevertheless, despite these limitations, the present study provides prospective evidence supporting the feasibility of integrating patient-specific three-dimensional planning into a structured anatomy-driven decision-support algorithm. Rather than replacing surgical judgement, the Vall d’Hebron 3D-PPS Algorithm offers an objective framework capable of standardising multidisciplinary evaluation, facilitating patient-specific surgical planning and improving consistency in surgical approach selection for complex parapharyngeal space lesions.

## 5 Conclusion

Patient-specific three-dimensional virtual planning provides clinically relevant anatomical information that complements conventional imaging and supports more informed surgical planning for parapharyngeal space lesions. Its value lies not only in improving anatomical visualisation but also in facilitating structured multidisciplinary decision-making and surgical preparedness. By integrating patient-specific three-dimensional reconstruction with detailed endoscopic anatomical knowledge, the Vall d’Hebron 3D-PPS Algorithm provides a reproducible anatomy-driven framework for preoperative surgical planning. These prospective findings support the feasibility and potential clinical utility of this approach; however, further multicentre studies are needed to validate its reproducibility and determine its impact on clinical outcomes before broader adoption can be recommended.

## Data Availability

The original contributions presented in the study are included in the article/**Supplementary Material**. Further inquiries can be directed to the corresponding author.
